# Rescheduling trains by crossover tracks to promote service quality of urban rail transit under partial blockages

**DOI:** 10.1371/journal.pone.0296018

**Published:** 2024-01-02

**Authors:** Yuxuan Long, Baoming Han, Weiteng Zhou, Zebin Chen, Ruixia Yang

**Affiliations:** 1 School of Traffic and Transportation, Beijing Jiaotong University, Beijing, China; 2 School of Systems Science, Beijing Jiaotong University, Beijing, China; Southwest Jiaotong University, CHINA

## Abstract

This study focuses on the rescheduling problem with disruptions that cause partial blockages in the urban rail transit (URT), contributing to extending the relative train rescheduling studies. The alternative driving measure (ADM), which could be regarded as one train rerouting measure, is used to skip the blocked section, and a mixed-integer nonlinear programming (MINLP) model is built based on it. Time-varying passenger flow as well as the turnaround process of rolling stocks is taken into consideration. To solve the model, a customized genetic algorithm is used to quickly generate high-quality solutions. Real-world data is studied and sensitivity analyses are taken to verify the feasibility and advantage of ADM. The results validate the proposed model and algorithm, as well as confirm that ADM shows significantly better performance than the practical operation measure in promoting passenger service quality of URT under partial blockage.

## Introduction

Urban rail transit (URT) has been developing at a great high speed these years, which is broadly accepted for its large capacity, high service frequency, low energy consumption, and low pollution [1, 2]. As a result of its high passenger density and short headway, any unexpected technical or human incident may cause heavy disturbances to passengers’ trips, pushing operators to focus on the passenger service [3, 4]. Cacchiani et al. [5] defined incidents as disturbances and disruptions in an overview paper, and rescheduling problems for them have been widely studied for decades and many achievements have been got.

Usually, there are several train rescheduling measures for the operator to choose from considering different situations. It is worth noting that though there are significant differences between URT systems and passenger railway systems, many rescheduling measures are similar for both systems, and researchers would use these measures for different systems by giving specific consideration to line conditions, technical standards, etc. Firstly, using the buffer time, which is also named retiming, could be regarded as an efficient way for real-time operation [6], which reschedules trains by adjusting the running time, dwell time, as well as the headway [7–9]. Besides retiming, flexible stopping, which is also called skip-stop, is a well-known rescheduling measure that can speed up the circulation of trains and reduce stranded passengers [10, 11]. Both mentioned measures mainly focus on incidents that do not cause any blocked section in the line, while some disruptions could result in partial or complete blockages [12, 13]. As a substitute measure, bus bridging is gradually contended to be one important part of disruption management, which refers to using buses to transport passengers to their planned destination during metro disruption [14, 15]. Bus bridging shows a positive influence on relieving stranded passengers caused by disruption, but the passenger service quality inside the urban railway system could not be improved by this measure.

In previous studies which focus on rescheduling during a complete or partial blockage, the rerouting measure has been popular, which could be divided into passenger rerouting and train rerouting. Passenger rerouting means that passengers transfer to other trains or choose other routes to continue their journey to ensure they could reach their destinations during the disruption [16, 17], which is mainly used in railway rescheduling or metro network rescheduling problems. Train rerouting could be used for both a network and a line [18, 19], and for a line it contains two kinds of measures. One of them is short-turning, which has been widely studied in both railway and URT. Ghaemi et al. [20] considered short-turning trains in one direction at multiple stations adjacent to the blocked section, and Ghaemi et al. [21] investigated the impact of the disruption length based on the rescheduling measure which was short-turning. In URT, most of the rescheduling studies with the rerouting measure have used short-turning. Bešinović et al. [1] proposed an integrated traffic management model for the complete blockages in urban railway lines, and Long et al. [16] studied the passenger-oriented rescheduling of trains and rolling stocks under disruption for a metro network. Li et al. [22] considered the short-turning to be one possible choice of the train turnaround and focused on the the resilience of urban rail systems. Short-turning could ensure the balance of the rolling stock and help improve service quality in cases of complete blockages. However, considering the partial blockage, passengers who want to travel for a long-distance crossing the blocked section can only wait for recovery with short-turning, which will significantly increase the travel time of these passengers, resulting in a decrease in passenger satisfaction. Worse yet, if these passengers are stranded for a relatively long time, they would prefer to choose other transportation modes to get to their destinations, which causes a significant decline in the service quality of URT.

The alternative driving measure (ADM), which is the other measure of train rerouting, shows significant strength in dealing with partial blockages by offering an efficient way to let passengers continue their trips during the disruption [23, 24]. Though very few, there are some scholars who have paid attention to taking ADM into account in the train rescheduling problem. Xu et al. [25] proposed a model with ADM during the partial blockage and verified the advantage of this measure in declining the delay of train services and ensuring the service balance at two terminals. However, their research didn’t take passengers into account, while ADM could bring significant improvement in passenger service quality. Huang et al. [26] developed rescheduling models based on two strategies considering the partial blockage of the circular line, focusing on alleviating the inconvenience for passengers and regaining the nominal train regularity. One of the two used strategies in this research was the same as ADM. However, they didn’t consider the rolling stock circulation, which is important under partial blockages, because it is difficult for rolling stocks in the blocked direction to finish their task and begin the new in time. Also, in their future research part, they mentioned one may extend those models to use the actual numbers of boarding and alighting passengers at each station, as they had done some simplification.

It is widely accepted that the train rescheduling problem with a large number of integer variables is challenging to get an optimal solution in acceptable solving time. Therefore, many researches applied heuristic algorithms as well as metaheuristics to get near-optimal solutions for the train rescheduling problems. Zhu et al. [27] studied the collaborative optimization of rescue operation and timetable rescheduling problem and solved the proposed model by a tabu search algorithm. Simulated annealing algorithm was used in Tamannaei et al. [28] for tackling the large-scale train rescheduling problems. Xu et al. [29] designed an efficient genetic algorithm to solve a passenger-oriented model for train rescheduling, and Han et al. [30] designed an algorithm based on the Non-dominated Sorting Genetic Algorithms-II (NSGA-II) to find the trade-off between two objectives of the proposed model. Zhang et al. [31] designed an iterative meta-heuristic for the general metro rescheduling algorithm to classify an accident and determine rescheduling solutions. For metaheuristics to solve the train rescheduling problem, some studies combined the rescheduled timetable with some other operation plans to be designed as the initial solution [29, 30], while some other studies may have the operation plan to be the initial solution and use solvers such as Gurobi to get the rescheduled timetable [27]. Then the iteration process would be taken to generate relative and better solutions, and finally find one near-optimal solution for the problem with some terminal conditions.

Table 1 shows the comparison of different publications of related rescheduling models and methods. For the names of columns, the time-varying passenger flow with OD is indicated by TPOD, and the turnaround process is represented by TP.

**Table 1:** The comparison of different publications of related rescheduling models and methods.

Publication	Transportation Mode	Blockage	Model	Strategy	TPOD	TP	Solution Method
Zhang et al. [8]	Urban railway/Metro	N	NLOC	RT	N	N	MPC
Xu et al. [29]	Urban railway/Metro	N	MINLP	RT	N	N	GA
Gao et al. [11]	Urban railway/Metro	N	MILP	FS	N	C	IA
Zhu et al. [27]	Urban railway/Metro	N	MILP	RT&FS	N	C	TS & Gurobi
Han et al. [30]	Railway	P	MILP	TR&PR	N	C	NSGA-II
Tamannaei et al. [28]	Railway	C	MILP	RT&TR	N	N	SA
Long et al. [16]	Urban railway/Metro	C	MILP	PR & ST	N	C	CPLEX
Ghaemi et al. [21]	Railway	C	MILP	PR & ST	N	C	Gurobi
Bešinović et al. [1]	Urban railway/Metro	C	MINLP	ST	C	C	IDM
Zhan et al. [24]	Railway	P	MILP	ADM	N	C	RH & CPLEX
Xu et al. [25]	Urban railway/Metro	P	MILP	ADM	N	N	CCA
Huang et al. [26]	Urban railway/Metro	P	MILP	ST & ADM	N	N	CPLEX
This paper	Urban railway/Metro	P	MINLP	ADM	C	C	Customized GA

**Blockage**: no blockage is considered in the publication (N); partial blockage is considered (P); complete blockage is considered (C).

**Model**: the nonlinear optimal control (NLOC); the mixed-integer nonlinear programming (MINLP); the mixed-integer linear programming (MILP).

**Strategy**: retiming (RT); flexible stopping (FS); train rerouting (TR); passenger rerouting (PR); short-turning (ST).

**TPOD & TP**: consider (C); non-consider (N).

**Solution Method**: model predictive control (MPC); the genetic algorithm (GA); the iterative algorithm (IA); the tabu algorithm (TS); the simulated annealing algorithm (SA); the integrated disruption management (IDM); the rolling horizon (RH); the capacity check algorithm (CCA).

In summary, there are seldom studies using ADM as the rescheduling strategy for partial blockage in URT. In this paper, we have studied normal urban railway lines, i.e., acyclic lines, which could be regarded as the subset of a network if only one disruption occurs at one time, and ADM has been taken into consideration to continue all the services during the disruption. Compared with the few existing papers that apply ADM, this paper has contributions in considering the time-varying passenger flow with OD, as well as the turnaround process of rolling stocks. Specifically, the contributions and differences of this paper are given as follows:

1. This paper considers dynamic passenger flows, which are dealt with as actual numbers with origins, destinations, as well as certain arrival times, instead of arrival rates and alighting ratios for simplicity. Also, besides waiting and boarding, passenger behaviors are extended in this paper by introducing leaving after waiting for a relatively long time. Both improvements in the passenger flow are not considered in previous research which studied ADM in URT.
2. This paper develops a mixed-integer nonlinear programming model based on the ADM, which takes the turnaround process into account. Though the turnaround process contains an important part of rolling stock circulation, previous research using ADM in URT almost ignores it to simplify the rescheduling problem. A customized genetic algorithm is designed to meet the computing requirement of rescheduling, of which the population generation could produce effective child individuals, and a check process is used to ensure individuals’ feasibility, which could help handle integer programming models with many complicated constraints by genetic algorithm.

The rest of this paper is structured as follows. In the Mathematical model section we describe the rescheduling problem and then propose a mixed-integer nonlinear model based on ADM. A customized genetic algorithm is designed in the Algorithm description section to solve the model, and in the Case study section we deal with the real-world data to validate the proposed model and algorithm, also verify the advantage of ADM in improving passenger service quality. At last, we deliver a conclusion of this paper and provide some further ideas in the Conclusions section.

## Mathematical model

### Problem description

#### Basic descriptions

**Fig 1:**
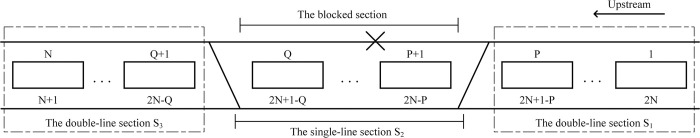
The section division of a line after the disruption.

As aforementioned, incidents causing partial blockage would divide a bidirectional urban railway line into two double-line sections and one single-line section by two crossovers. Take the upstream direction to be the blockage direction as an example, and Fig 1 shows the three sections named as *S*_1_, *S*_2_, and *S*_3_ respectively. Let the set of upstream-direction stations $S_{J}^{u}=\{1,2,\ldots ,N\}$, and the set of downstream-direction stations $S_{J}^{d}=\{N+1,N+2,\ldots ,2N\}$. During the disruption period, the three sections could be regarded as five subsets of stations according to the two crossovers on both sides of the blocked section, and $S_{1}^{u}=\{1,2,\ldots ,P\}$, $S_{1}^{d}=\{2N+1-P,2N+2-P\ldots ,2N\}$, $S_{2}=\{2N+1-Q,2N+2-Q,\ldots ,2N-P\}$, $S_{3}^{u}=\{Q+1,Q+2,\ldots ,N\}$, $S_{3}^{d}=\{N+1,N+2,\ldots ,2N-Q\}$. Station P represents the last station before the blocked section in the upstream direction, and station Q is the station at the end of the blocked section. The shape of crossovers shown in Fig 1 is opposite, but other shapes are also feasible for this paper, only affecting some parameters for trains to pass.

**Fig 2:**
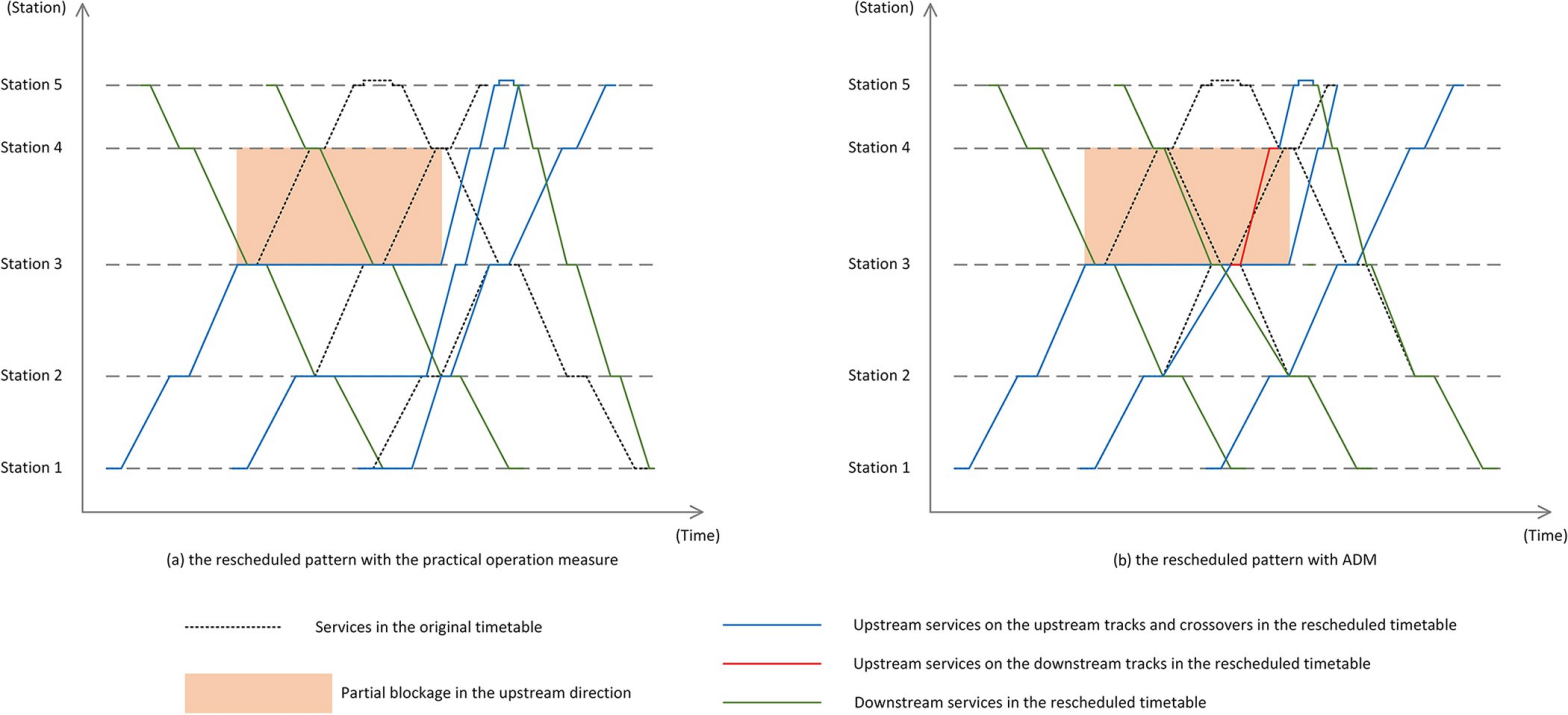
An illustration of two rescheduling strategies for their rescheduled pattern.

To the best of our knowledge, for the commonly used measure in real-life operations for partial blockages in China, impacted train services in the upstream direction are mostly asked to wait at stations before the blocked section during the disruption, leading to unbalance of rolling stock circulation as well as passenger unpleasantness [25]. After recovery, train services would be rescheduled by compressing the buffer time to decline delays, which is also called retiming, and finally achieve the original timetable. This commonly used measure is called *practical operation*, and *stranded train* is used in this paper to indicate the rolling stock stopping during the disruption. When the disruption occurs, train services in the upstream direction which have already passed station Q would not be affected, and it is similar for train services in the downstream direction which have already passed station 2N−Q. But for train services in the section between station P+1 and station Q, they would be stranded during the disruption. For these stranded trains, they are assumed to stop at the nearest station and wait for recovery. An example is given in Fig 2 to compare the rescheduling process of the two rescheduling measures, the practical operation measure and the ADM, in which the first train service in the upstream direction represented by the blue line is stranded at Station 3 due to the partial blockage. In the planned pattern of rolling stock circulation, the third train service in the downstream direction is connected with the first upstream train service. The blocked section in Fig 2 contains Station 3 and Station 4, as well as the section between them, and the crossovers are before Station 3 and after Station 4, respectively.

As shown in Fig 2(A), with the practical operation measure, all the blue lines which represent upstream train services would wait at Stations 3, 2 and 1 separately until the blockage ends, and passengers in the upstream direction also have to stop and wait for recovery. Moreover, the third downstream train service has to wait until the first upstream train service departs from Station 5 and finishes the turnaround task, because they share the same rolling stock and no backup rolling stock is taken into consideration. While considering the rescheduled pattern with ADM, Fig 2(B) shows that during the disruption, the second upstream train service could continue its journey from Station 2 to Station 5 by the crossovers and downstream tracks, which is shown in the red line. Passengers in the first upstream train who want to travel to Station 4 or 5 could alight at Station 3 and board the second upstream train at the opposite platform, instead of waiting in the stranded train until recovery. Also, the third train service in the downstream direction is connected with the second upstream train service now, and it means the third downstream train service could begin its journey earlier with ADM than that with the practical operation method. In this way, ADM could show a significant advantage in maintaining rolling stock circulation and ensuring passenger reachability during the disruption. However, because the region from Station 3 to Station 4 is shared by two-direction train services as a single-line section, the capacity over this region would be reduced with ADM [26]. Indeed, ADM is not suitable when the single-track section is too long, therefore, the blockage studied in this paper is considered to exist between two adjacent crossovers instead of a long range.

#### Descriptions of train services

In this paper, *train service* is defined as a rolling stock operating between two terminal stations. *f* is used as the index of train services in the upstream direction, and *g* is used for train services in the downstream direction. *F* and *G* represent the set of train services in each direction, respectively. *j* is used as the index of stations, $j\in S_{J}^{u}\cup S_{J}^{d}$, then *j*−1 and *j*+1 respectively indicate stations before and after station *j* along the train service direction. For train services in the upstream direction, there are two groups divided by whether the service passes the single-line section. Let *F*_*d*_ be the set of train services in the upstream direction that needs to pass the single-line section, *F*_*d*_ = {*f*_1_, *f*_2_,…,*f*_*k*_}, *F*_*d*_⊂*F*. Namely, *f*_1_ indicates the first train service in the upstream direction that needs to pass the single-line section and *f*_*k*_ is the last one. *f*_*d*_ is used as the index of train services that need to pass the single-line section, *f*_*d*_∈*F*_*d*_.

As aforementioned, the blockage doesn’t exist for a long range, meaning that there would not be too many train services stranded in the blocked section. Therefore, this paper takes the situation in which there is only one stranded train service *f*_0_ in the upstream direction stopping in the blocked section as an example. To find which service is the *f*_0_, we have

$$a_{f_{0,P+1}}^{o}\leq T_{0}$$ (1)

$$a_{f_{0,Q}}^{o}\leq T_{0}$$ (2)

$$a_{f_{0}-1,Q}^{o}\leq T_{0}$$ (3)

$$a_{f_{1},P}^{o}\leq T_{0}$$ (4)

where $a_{f_{0},P+1}^{o}$ is the arrival time of service *f*_0_ at station *P*+1 in the original timetable, and $a_{f_{0},Q}^{o},a_{f_{0}-1,Q}^{o}$ are similar; $d_{f_{1},P}^{o}$ is the departure time of service *f*_1_ at station *P* in the original timetable; *T*_0_ is the start time point of the blockage, and it is easy to find *f*_1_ = *f*_0_+1, namely, *f*_1_ is the first service practically behind *f*_0_. Formula (1) represents that *f*_0_ has already entered the blocked section when the disruption occurs, while it has not left this section according to Formula (2). Formula (3) & (4) indicate that there is only one train service stranded due to the disruptions. *j*_0_ is used to represent the last station served by *f*_0_, and for $j_{0}\in \{P+1,P+2,\ldots ,Q\},a_{f_{0},j_{0}}^{o}\leq T_{0}\leq a_{f_{0},j_{0}+1}^{o}$. Correspondingly, we use *g*_0_ to represent the last train service in the downstream direction to leave station 2*N*−*Q* before the disruption, and then *g*_1_ indicates the first train service in the downstream direction that needs to pass the single-line section. For train services in the downstream direction we have

$$d_{g_{0},2N-Q}^{o}\leq T_{0}$$ (5)

$$d_{g_{1},2N-Q}^{o}\leq T_{0}$$ (6)

We use *G*_*s*_ = {*g*_1_, *g*_2_,…,*g*_*h*_} to be the set of affected services in the downstream direction which need to pass the single-line section during the disruption, and *g*_*s*_ is used to represent the index of these train services, *g*_*s*_∈*G*_*s*_.

#### Descriptions of rolling stock circulation

Considering the circulation of rolling stocks, it is important to ensure whether there is any backup rolling stock in the depot, and this would have a significant influence on the rescheduling process. Obviously, more backup rolling stocks could make the rescheduling problem under the partial blockage much easier. While in this paper, we take the limited amount of rolling stocks into consideration and only focus on the existing rolling stocks in the planned pattern, which could be a difficult but possible situation. There has already been a planned pattern for the connections of rolling stocks, which are described as given binary variables $\delta_{f,g}^{o}$ and $\delta_{g,f}^{o}$. There is $\delta_{f,g}^{o}=1$ if service *g* in the downstream direction uses the same rolling stock of service *f* in the upstream direction, and $\delta_{g,f}^{o}=1$ means service *f* in the upstream direction uses the same rolling stock of service *g* in the downstream direction. Correspondingly, binary variables *δ*_*f*,*g*_ and *δ*_*g*,*f*_ are used to describe the connections of rolling stocks in the rescheduled pattern.

As the disruption occurs in the upstream direction, during the whole rescheduling period there is always $\delta_{g,f}=\delta_{g,f}^{o}$. Whether there is any difference between $\delta_{f,g}^{o}$ and *δ*_*f*,*g*_ depends on the number of stranded trains caused by the blockage, and in the studied case with one stranded train, during the disruption there is $\delta_{f_{1},g}=\delta_{f_{0},g}^{o},\delta_{f_{2},g}=\delta_{f_{1},g}^{o}$, …, $\delta_{f_{k},g}=\delta_{f_{k-1},g}^{o}$, and *g*∈*G*. Then after the recovery, *f*_0_ would continue its service at remained stations, $\delta_{f_{0},g}=\delta_{f_{k},g}^{o}$, and $\delta_{f_{k}+1,g}=\delta_{f_{k}+1,g}^{o}$, …, $\delta_{f_{\left|F\right|},g}=\delta_{|F|,g}^{o}$.

*Remark*. For the situation in which there is no train service stranded in the blocked section, it could be regarded as one simplified situation of the studied case. For all train services *f*∈*F*, *g*∈*G*, $\delta_{g,f}=\delta_{g,f}^{o}$ and $\delta_{f,g}=\delta_{f,g}^{o}$. While for situations with more than one stranded train service, the rescheduling pattern for the connection of rolling stocks could be projected with the corresponding index, for example, $\delta_{f_{2},g}=\delta_{f_{0},g}^{o}$ for two stranded trains, etc. All the situations mentioned have a similar rescheduling process except the pattern for the rolling stock circulation, and then we take the situation with one stranded train service as the case to be studied.

#### Descriptions of passengers

**Fig 3:**
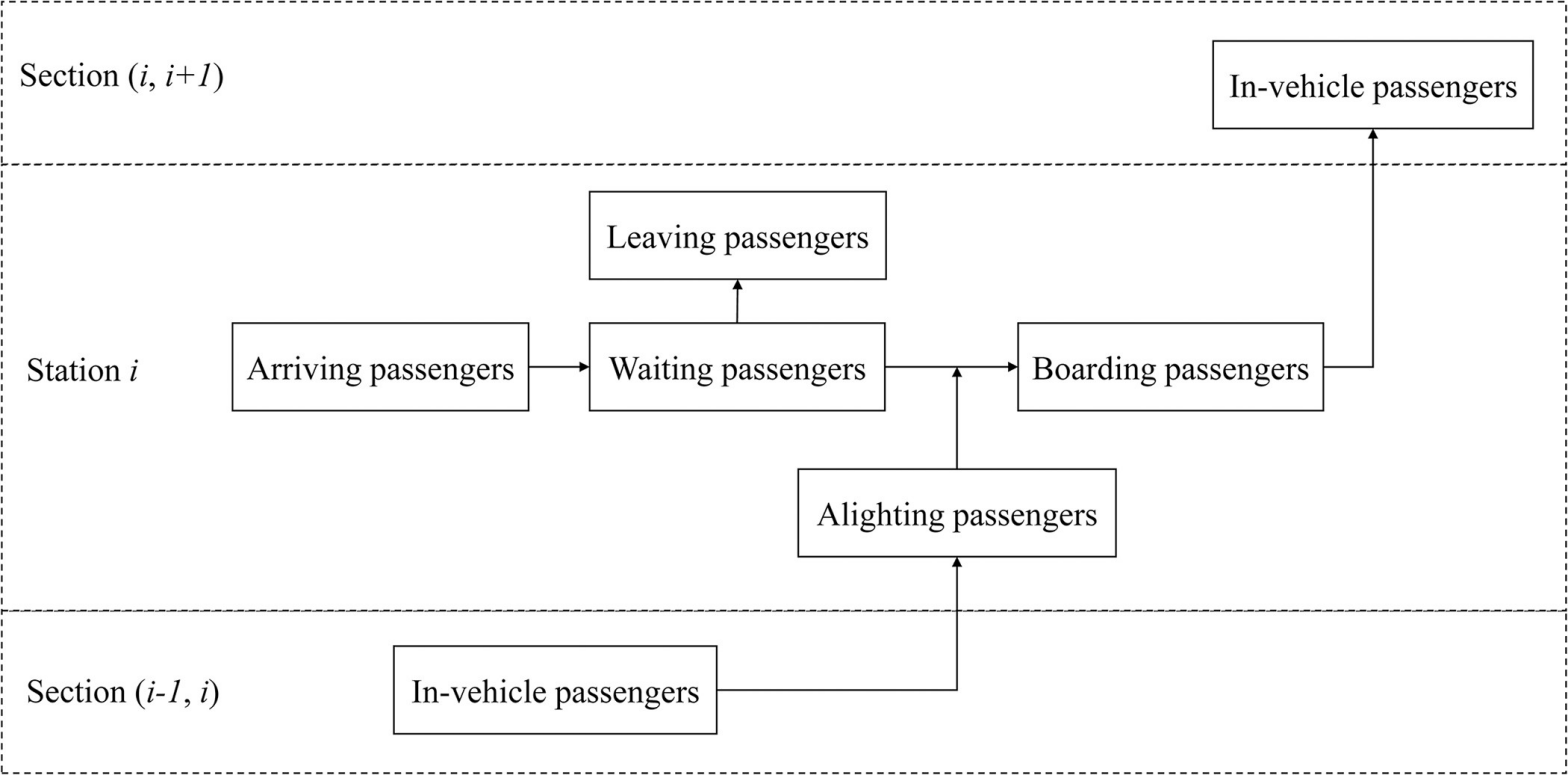
Procedures of passenger behaviors.

Procedures of passenger behaviors considered in this paper include arriving, waiting or leaving, boarding, and alighting, which is shown in Fig 3. Parameter $p_{t}^{jj^{\prime }}$ represents the number of passengers that arrive at station *j* at time point *t* with the destination station *j*′, and $p_{t}^{jj^{\prime }}=0$ when station *j* and *j*′ could not form an OD pair. However, during the disruption, some OD pairs would be affected due to the blockage, in which there is at least one station, origin or destination, in the blocked section. For passengers who arrive at or expect to travel to station *j* in the blocked section during the disruption, *j* in their OD pairs should be replaced by 2N+1−*j*, which means the opposite platform of the same station.

Before the service arrives, there could probably be some leaving passengers because the waiting time is too long for them. Integer variable $q_{i}^{j}$ is used to describe the number of passengers leaving station *j* for other transportation modes before service *i* arrives at this station, and $P_{i}^{j}$ indicates the number of passengers finally board service *i* at station *j*. A parameter *t*^*O*^ is given as a constant which depends on the results of a simple survey for the tolerance time for passengers to wait for one service in URT, which is related to $q_{i}^{j}$. Moreover, binary variable $z_{it}^{j}=1$ indicates that passengers arriving at station *j* at the time point *t* finally could board service *i*. In this paper, we take the off-peak period and the leaving behavior into consideration, and the limitation of vehicle capacity is ignored, meaning that whether passengers could board one service only depends on the time factor.

### Problem assumptions

For rigour and simplicity, several assumptions are made for the problem as follows:

1. The number of used rolling stocks is a constant, namely, the disruption does not affect the technical condition of all rolling stocks and no backup rolling stock would be considered in the rescheduling process. Tracks and stations are all allowable for train services from both directions, and there is no difference among rolling stocks.
2. The stranded train would stop at the last served station during the disruption. Passengers on the stranded train would alight and go to the opposite platform for another service. Also, passengers who expected to arrive at the blocked station during the disruption would practically wait at the opposite platform to board one train service.
3. Disruptions occur in off-peak hours, and all passengers who have not left could board the first train service after their arrival without waiting for the next one. Passengers would choose to leave the URT for another transportation mode when the waiting time is longer than their tolerance time.

### Model formulations

Based on problem description and problem assumptions, model formulations are given as notations, timetable and passenger flow formulations, as well as objective functions.

#### Notations

All the relevant notations and parameters used in formulating the model are shown in Table 2.

**Table 2:** Notations and parameters involved in formulating the model.

Sets	Description
$S_{J}^{u}$	Set of stations in the upstream direction
$S_{J}^{d}$	Set of stations in the downstream direction
$S_{1}^{u}/S_{1}^{d}/S_{2}/S_{3}^{u}/S_{3}^{d}$	Subsets of stations during the disruption according to Fig 1
*F*	Set of train services in the upstream direction, |*F*| is the total number of train services in the upstream direction
*G*	Set of train services in the downstream direction, |*G*| is the total number of train services in the downstream direction
*F*_*d*_	Set of train services in the upstream direction that need to pass the single-line section during the disruption, *F*_*d*_⊂*F*
*G*_*s*_	Set of train services in the downstream direction that need to pass the single-line section during the disruption, *G*_*s*_⊂*G*
Indices	Definition
*j*	Index of stations, $j\in S_{J}^{u}\cup S_{J}^{d}$
*N*	Index of the last station in the upstream direction
*P*	Index of the last station before the blocked section in the upstream direction
*Q*	Index of the last station of the blocked section in the upstream direction
*f*	Index of train services in the upstream direction, *f*∈*F*
*g*	Index of train services in the downstream direction, *g*∈*G*
*f*_*d*_	Index of train services in the upstream direction that need to pass the single-line section during the disruption, *f*_*d*_∈*F*_*d*_
*g*_*s*_	Index of train services in the downstream direction that need to pass the single-line section during the disruption, *g*_*s*_∈*G*_*s*_
*f*_1_	Index of the first train service in the upstream direction that needs to pass the single-line section
*f*_*k*_	Index of the last train service in the upstream direction that needs to pass the single-line section
*f*_0_	Index of the stranded train in the upstream direction
*g*_1_	Index of the first train service in the downstream direction that needs to pass the single-line section
*g*_*h*_	Index of the last train service in the downstream direction that needs to pass the single-line section
*g*_0_	Index of the last train service in the downstream direction to leave station 2*N*−*Q* before the disruption
Parameters	Definition
$d_{f,j}^{o}/a_{f,j}^{o}$	Departure or arrival time of service *f* at station *j* in the original timetable
$d_{g,j}^{o}/a_{g,j}^{o}$	Departure or arrival time of service *g* at station *j* in the original timetable
$w_{j}^{min}$	Minimum dwell time for train services at station *j*
$r_{j-1}^{min}/r_{j-1}^{max}$	Minimum/maximum running time for train services between station *j*−1 and station *j*
$\delta_{g,f}^{o}$	Given binary value, $\delta_{g,f}^{o}=1$ if service *f* in the upstream direction uses the same rolling stock of service *g* in the downstream direction
$\delta_{f,g}^{o}$	Given binary value, $\delta_{f,g}^{o}=1$ if service *g* in the downstream direction uses the same rolling stock of service *f* in the upstream direction
*δ*_*g*,*f*_	Binary value, *δ*_*g*,*f*_ = 1 if service *f* in the upstream direction uses the same rolling stock of service *g* in the downstream direction
*δ*_*f*,*g*_	Binary value, *δ*_*f*,*g*_ = 1 if service *g* in the downstream direction uses the same rolling stock of service *f* in the upstream direction
*t*_*c*_	Additional time for train services to pass the crossover
$t_{min}^{b}$	Minimum time for one rolling stock to finish the turnaround task
$p_{t}^{jj^{\prime }}$	Number of passengers that arrive at station *j* at time point *t* with the destination station *j*′
*t* ^*O*^	Tolerance time of passengers
*h*_1_	Minimum headway for two consecutive train services to arrive at or depart from one station
*h*_2_	Minimum headway for the following service to arrive at a station that the leading one departed from
*t*_*s*_	Security headway for train services in different directions to enter the single-track section alternately
*T*_0_	The start time point of blockage
*T*_1_	The end time point of blockage
*t*_*p*_	Penalty time for leaving passengers
*μ*	Coefficient of passenger feeling sensitivity
Decision variables	Definition
*d*_*f*,*j*_/*a*_*f*,*j*_	Departure or arrival time of service *f* at station *j* in the rescheduled timetable
*d*_*g*,*j*_/*a*_*g*,*j*_	Departure or arrival time of service *g* at station *j* in the rescheduled timetable
*w*_*f*,*j*_/*w*_*g*,*j*_	Actual dwell time of service *f* or *g* at station *j*
*r*_*f*,*j*_/*r*_*g*,*j*_	Actual running time of service *f* or *g* between station *j* and *j*+1
*x*_*f*,*g*_/*x*_*g*,*f*_	Binary variable, *x*_*f*,*g*_/*x*_*g*,*f*_ = 1 if service *g*/*f* enters the single-line section when service *f*/*g* in the opposite direction leaves the section
$t_{f}^{b}/t_{g}^{b}$	Actual time of service *f* or *g* for turnaround
$q_{f}^{j}/q_{g}^{j}$	Number of passengers leaving station *j* for other transportation modes before service *f*/*g* arrives at this station
$P_{f}^{j}/P_{g}^{j}$	Number of passengers finally board service *f*/*g* at station *j*
*ω*	Ratio of failed travel for passengers
$z_{f,t}^{j}/z_{g,t}^{j}$	Binary variable, $z_{f,t}^{j}/z_{g,t}^{j}=1$ if passengers arriving at station *j* at the time point *t* finally could board service *f*/*g*

#### Timetable formulations

1. Arrival and departure formulations

In the rescheduled timetable, let *a*_*g*,*j*_ and *d*_*g*,*j*_ be the actual arriving and departure time at station *j* of service *g* in the downstream direction, there is:

$$d_{g,f}=a_{g,j}+w_{g,j},\forall g\in G,j\in S_{J}^{d}$$ (7)

$$a_{g,j}=d_{g,j-1}+r_{g,j-1},\forall g\in G,j\in S_{J}^{d}\backslash \{N+1\}$$ (8)

where *w*_*g*,*j*_ is the actual dwell time of service *g* at station *j*, and *r*_*g*,*j*−1_ is the actual running time of service *g* from station *j*−1 to station *j*. Considering the practical operation requirement, there are limitations on the dwell time and running time:

$$w_{g,j}\geq w_{j}^{\min},\forall g\in G,j\in S_{J}^{d}$$ (9)

$$r_{j-1}^{\min}\geq r_{g,j-1}\leq r_{j-1}^{\max},\forall g\in G,j\in S_{J}^{d}\backslash \{N+1\}$$ (10)

where $w_{j}^{min}$ is the minimum dwell time at station *j*; $r_{j-1}^{min}$ and $r_{j-1}^{max}$ represent the limitations of running time between station *j*−1 and station *j*. In fact, the actual dwell time and running time would depend on the operation instructions and the limitations.

For train services in the upstream direction, there is:

$$d_{f,j}=a_{f,j}+w_{f,j},\forall f\in F,j\in S_{J}^{u}$$ (11)

$$a_{f,j}=d_{f,j-1}+r_{f,j-1},\forall f\in F\backslash F_{d},j\in S_{J}^{u}\backslash \{1\}$$ (12)

$$a_{f_{d},j}=d_{f_{d},j+1}+r_{f_{d},j+1},\forall f_{d}\in F_{d},j\in S_{2}\backslash \{2N-P\}$$ (13)

$$a_{f_{d},j}=d_{f_{d},j-1}+r_{f_{d},j-1},\forall f_{d}\in F_{d},j\in S_{1}^{u}\cup S_{3}^{u}\backslash \{1,Q+1\}$$ (14)

$$a_{f_{d},2N-P}=d_{f_{d},P}+r_{f_{d},P}+t_{c},\forall f_{d}\in F_{d}$$ (15)

$$a_{f_{d},Q+1}=d_{f_{d},2N+1-Q}+r_{f_{d},Q}+t_{c},\forall f_{d}\in F_{d}$$ (16)

where *w*_*f*,*j*_ is the actual dwell time of service *f* at station *j*; *r*_*f*,*j*−1_ is the actual running time of service *f* from station *j*−1 to station *j*; *t*_*c*_ is the additional time of trains to pass the crossover and Eqs (13)—(16) describe the process for train services *f*_*d*_ entering and leaving the single-line section by the crossover.

Especially, considering the stranded train *f*_0_ and the last train service passing the single-line section *f*_*k*_, we have

$$d_{f_{0},j_{0}}\geq T_{1}$$ (17)

$$d_{f_{k},P}<T_{1}$$ (18)

$$d_{f_{k+1},P}\geq T_{1}$$ (19)

where *T*_1_ is the end time point of blockage.

It is particular for the arrival times of train services at the first station of each direction, as there are two possible cases. Take services in the upstream direction as an example, as services in the downstream direction are similar. Some of services in the upstream direction use rolling stocks which are not from services in set *G*, as their rolling stocks correspond to earlier services in the downstream direction which are not considered in the rescheduling process. Their arrival times at station 1 could be the same as the original timetable. While others which are connected with services *g*∈*G*, the calculation should consider both the turnaround process and the departure time of service *g* at station 2*N*. Therefore, the arrival time of train service at the first station of each direction could be computed by

$$a_{f,1}=\sum_{g\in G}\delta_{g,f}\cdot (d_{g,2N}+t_{g}^{b})+(1-\sum_{g\in G}\delta_{g,f})\cdot a_{f,1}^{o},\forall f\in F$$ (20)

$$a_{g,N+1}=\sum_{f\in F}\delta_{f,g}\cdot (d_{f,N}+t_{f}^{b})+(1-\sum_{f\in F}\delta_{f,g})\cdot a_{g,N+1}^{o},\forall g\in G$$ (21)

$$\sum_{g\in G}\delta_{g,f}\leq 1,\forall f\in F$$ (22)

$$\sum_{f\in F}\delta_{f,g}\leq 1,\forall g\in G$$ (23)

where $t_{g}^{b}$ is the time for the rolling stock of train service *g* to finish the turnaround task, $t_{f}^{b}$ is the time for the rolling stock of train service *f* to finish the turnaround task, and both of them should satisfy the minimum turnaround time for rolling stocks, i.e.,

$$t_{g}^{b}\geq t_{\min}^{b},\forall g\in G$$ (24)

$$t_{f}^{b}\geq t_{\min}^{b},\forall f\in F$$ (25)

2. Headway formulations

To ensure the security of operation, consecutive train services should follow several headway limitations. Then we formulate the headway constraints for train service *g* in the downstream direction as

$$d_{g,j}\geq d_{g-1,j}+h_{1},\forall g\in G,j\in S_{J}^{d}$$ (26)

$$a_{g,j}\geq d_{g-1,j}+h_{2},\forall g\in G,j\in S_{J}^{d}$$ (27)

where *h*_1_ is the minimum headway for two consecutive train services to depart from one station; *h*_2_ is the minimum headway for the following service to arrive at a station that the leading one departed from; *d*_*g*−1,*j*_ is used to represent the time point of the last train service departed from station *j* before service *g*, and when *g* = 1, *d*_*g*−1,*j*_ could be gotten from the original timetable.

Then for train services *f* in the upstream direction, there are

$$d_{f,j}\geq d_{f-1,j}+h_{1},\forall f\in F,j\in S_{J}^{u}\cup S_{2}$$ (28)

$$a_{f,j}\geq d_{f-1,j}+h_{2},\forall f\in F,j\in S_{J}^{u}\cup S_{2}$$ (29)

where *d*_*f*−1,*j*_ is used to represent the time point of the last train service departed from station *j* before service *f*. Also, when *f* = 1, *d*_*f*−1,*j*_ could be gotten from the original timetable.

3. Alternative driving formulations
  As aforementioned, train services in the upstream direction which need to pass the single-track section during the disruption are indicated by *f*_*d*_∈*F*_*d*_, and *F*_*d*_ = {*f*_1_, *f*_2_,…,*f*_*k*_}. Also, *g*_*s*_∈*G*_*s*_ correspondingly indicates services in the downstream direction. To ensure the security, there could not be both directions train services in the single-line section at the same time. Binary variables $x_{g_{s},f_{d}}$ and $x_{f_{d},g_{s}}$ represent orders for train services of entering the single-line section. $x_{g_{s},f_{d}}=1$ means that after *g*_*s*_ leaving the single-line section, it is *f*_*d*_ in the opposite direction to be the next one entering the single-line section, and $x_{f_{d},g_{s}}=1$ is similar. Then we have

$$d_{f_{d},P}\geq x_{g_{s},f_{d}}\cdot (d_{g_{s},2N-P}+t_{s}),\forall f_{d}\in F_{d},g_{s}\in G_{s}$$ (30)

$$d_{g_{s},2N-Q}\geq x_{f_{d},g_{s}}\cdot (d_{f_{d},2N-Q+1}+t_{s}),\forall f_{d}\in F_{d},g_{s}\in G_{s}$$ (31)

$$x_{g_{s},f_{d}}+x_{f_{d},g_{s}}=1,\forall f_{d}\in F_{d},g_{s}\in G_{s}$$ (32)

where *t*_*s*_ represents the security headway for train services in different directions to enter the single-track section. After *f*_*k*_ and *g*_*h*_, all train services run on the planned tracks due to the recovery of blockage. It is easy to find that in Eqs (30)—(31), there are multiplication of variables, namely, these equations are nonlinear.

#### Passenger flow formulations

There are several variables about the passenger flow as introduced in **Problem Description**, and they are all connected with the rescheduled timetable. Take service *g* as an example, and for service *f* the formulation is similar. *t*^*O*^ indicates the tolerance time of passengers, then for passengers arriving between services *g*−1 and *g*, when $a_{g,j}-d_{g-1,j}>t^{O}$, there is $z_{g,t}^{j}=1$ if ${t\geq a}_{g,j}-t^{O}$; if $a_{g,j}-d_{g-1,j}\leq t^{O},z_{g,t}^{j}=1$ is suitable for all passengers arriving at time point *t* for service *g* at station *j*. Eqs (33)–(34) show the relationship.

$$z_{g,t}^{j}=\{\begin{array}{c} 1,t\geq \max\{a_{g,j}-t^{o},d_{g-1,j}\}\\ 0,t<\max\{a_{g,j}-t^{o},d_{g-1,j}\} \end{array},\forall g\in G\backslash \{1\}$$ (33)

$$z_{1,t}^{j}=\{\begin{array}{c} 1,t\geq \max\{a_{g,j}-t^{o},T_{1}\}\\ 0,t<\max\{a_{g,j}-t^{o},T_{1}\} \end{array}$$ (34)

where $z_{g,t}^{j}$ is the binary variable to show whether passengers arriving at station *j* at the time point *t* finally could board service *g*.

With $z_{g,t}^{j}$, we could formulate the number of passengers boarding service *g* and those leaving before service *g* as follows

$$P_{g}^{j}=\sum_{t=d_{g-1,j}}^{d_{g,j}}\left(z_{g,t}^{j}\cdot \sum_{j'\in S_{J}^{d}}p_{t}^{jj'}\right),\forall g\in G,j\in S_{J}^{d}$$ (35)

$$q_{g}^{j}=\sum_{t=d_{g-1,j}}^{d_{g,j}}\sum_{j'\in S_{J}^{d}}p_{t}^{jj'}-P_{g}^{j},\forall g\in G,j\in S_{J}^{d}$$ (36)

As aforementioned, when *g* = 1, *d*_*g*−1,*j*_ could be gotten from the original timetable. Then for the total number of leaving passengers, we have

$$Q_{l}=\sum_{f\in F}\sum_{j\in S_{J}^{u}\cup S_{2}}q_{f}^{j}+\sum_{g\in G}\sum_{j\in S_{J}^{d}}q_{g}^{j}$$ (37)

Considering the leaving behavior of passengers, we use a variable *ω* to indicate the ratio of failed travel for passengers, namely, they could not board one service and have to leave the URT system. And for this ratio there is

$$\omega =Q_{l}/(Q_{l}+\sum_{f\in F}\sum_{j\in S_{J}^{u}\cup S_{2}}P_{f}^{j}+\sum_{g\in G}\sum_{j\in S_{J}^{d}}P_{g}^{j})$$ (38)

#### Objective functions

In this paper, passenger service quality is the total objective, and passenger cost is used to describe it. It is clear that the minimum passenger cost corresponds to the best passenger service quality, and the passenger cost could be considered by two parts due to whether passengers could board one service. For those who leave the URT system due to a relatively long waiting time, a parameter *t*_*p*_ is given as the penalty time to calculate their costs.

Considering passengers who finally board one service instead of leaving, the time cost including average waiting and travel time is taken into account first. The average waiting time for passengers is widely accepted to analyze the service quality of URT, and the average travel time also has an important influence on passengers’ traveling experience because people usually hope to arrive at their destination as soon as possible. For the sum of waiting time and travel time of all passengers there are:

$$\begin{array}{c} C_{tw}=\sum_{f\in F}\sum_{j\in S_{J}^{u}\cup S_{2}}\sum_{t=d_{f-1,j}}^{d_{f,j}}\left((d_{f,j}-t)\cdot z_{f,t}^{j}\cdot \sum_{j'\in S_{J}^{u}\cup S_{2}}p_{t}^{jj'}\right)\\ +\sum_{g\in G}\sum_{j\in S_{J}^{d}}\sum_{t=d_{g-1,j}}^{d_{g,j}}\left((d_{g,j}-t)\cdot z_{g,t}^{j}\cdot \sum_{j'\in S_{J}^{d}}p_{t}^{jj'}\right) \end{array}$$ (39)

$$\begin{array}{c} C_{ti}=\sum_{f\in F}\sum_{j\in S_{J}^{u}\cup S_{2}}\sum_{t=d_{f-1,j}}^{d_{f,j}}\left(z_{f,t}^{j}\cdot \sum_{j'\in S_{J}^{u}\cup S_{2}}\left((a_{f,j'}-d_{f,j})\cdot p_{t}^{jj'}\right)\right)\\ +\sum_{g\in G}\sum_{j\in S_{J}^{d}}\sum_{t=d_{g-1,j}}^{d_{g,j}}\left(z_{g,t}^{j}\cdot \sum_{j'\in S_{J}^{d}}\left((a_{g,j'}-d_{g,j})\cdot p_{t}^{jj'}\right)\right) \end{array}$$ (40)

Eqs (39)–(40) are nonlinear with variables to be the bounds of summary, and there are also multiplications of variables in these two equations. Then for the average waiting time and travel time of each passenger, we have

$$c_{tw}=c_{tw}/(\sum_{f\in F}\sum_{j\in S_{J}^{u}\cup S_{2}}P_{f}^{j}+\sum_{g\in G}\sum_{j\in S_{J}^{d}}P_{g}^{j})$$ (41)

$$c_{ti}=c_{ti}/(\sum_{f\in F}\sum_{j\in S_{J}^{u}\cup S_{2}}P_{f}^{j}+\sum_{g\in G}\sum_{j\in S_{J}^{d}}P_{g}^{j})$$ (42)

Besides the traditional time cost, there is special cost of service deviation. The deviation of the rescheduling service pattern from the original one was mainly regarded as the cost of the company in previous research because it means more operation consumption. However, with the development of the Passenger Information System (PIS), passengers can get enough information in advance and have expectations about the time for services to arrive at or depart from one station. And when their expectations are not realized, they may feel uncomfortable. That is why this paper put the deviation of service time as one influential factor in passenger benefit. Since early arrivals and departures could be allowed in the URT, the total service deviation of the rescheduling pattern from the original one is given by

$$\begin{array}{c} c_{d}=\sum_{f\in F}\sum_{j\in S_{J}^{u}\cup S_{2}}\left|a_{f,j}-a_{f,j}^{o}\right|+\sum_{f\in F}\sum_{j\in S_{J}^{u}\cup S_{2}}\left|d_{f,j}-d_{f,j}^{o}\right|\\ +\sum_{g\in G}\sum_{j\in S_{J}^{d}}\left|a_{g,j}-a_{g,j}^{o}\right|+\sum_{g\in G}\sum_{j\in S_{J}^{d}}\left|d_{g,j}-d_{g,j}^{o}\right| \end{array}$$ (43)

With the absolute value operation, Eq (43) is also nonlinear. For each passenger, he or she would only board at most one service at one time without transfer, and we have the average deviation as

$$c_{d}=C_{d}/(|F|+|G|)$$ (44)

$$\{\begin{array}{c} \min\;c=t_{p}\cdot \omega +(1-\omega )\cdot (\mu \cdot c_{d}+c_{tw}+c_{ti})\\ s.t.\;Formulations(1)-(44) \end{array}$$ (45)

where *c* represents the passenger cost, *ω* indicates the possibility for passengers to leave the URT, and the value of *t*_*p*_ could be gotten from the practical operation of one specific line based on experience. Due to the nonlinear characteristics of objective functions as well as Eqs (30)—(31), the proposed model (45) is a mixed-integer nonlinear programming model which is difficult to be solved by existing commercial solvers, and we have designed a customized GA to get the near-optimal solution in acceptable computing time.

### Algorithm description

To meet the requirement of real-time rescheduling, efficient algorithms are required, such as MPC from the control-oriented, heuristic algorithms like the genetic algorithm (GA), etc. Heuristic algorithms are widely used due to the large scale of rescheduling problems, and a customized GA is designed in this paper to solve the proposed model, which could be more adaptable for this train rescheduling problem. Compared with the standard one, the customized GA could get an optimized solution in a short time.

1. Population generation

**Fig 4:**
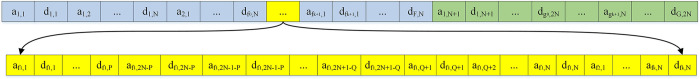
The coded chromosome of each individual.

For each individual, the chromosome is composed of two parts, which are coded by service times in the two directions separately, as shown in Fig 4. In this figure, cubes in blue represent times of services in the upstream direction without passing the single-line section, while yellow cubes represent those passing the single-line section in the upstream directions, as there are differences in the indexes of stations. Green cubes represent times of services in the downstream directions. All the cubes could be regarded as genes on each chromosome.

In the initialization process, solutions, which are shown as timetables of the rescheduling period, are generated by operating train services in chronological order under the limitation of traffic constraints shown in Section ‘Timetable formulations’. In this way, the solutions of the initial group would be all feasible. Differences among feasible solutions are mainly formed by the flexible running time of each section and dwell time at each station, as well as the different turnaround time of each service. In the standard GA, the initialization process has more randomness in each individual which represents the rescheduled timetable, because train services could stop and wait as long as the operator requests. However, the large buffer time could cause large delay for the later train services of the solutions, which would result in worse solution quality. The customized algorithm sets upper bounds on dwell times and turnaround times to ensure the quality of these feasible solutions, and these bounds are calculated by adding some buffer time for the corresponding time in the original timetable.

The fitness *f*_*i*_ of each individual in one generation is calculated by the formulation (46), and roulette wheel selection is applied to select the parents. Let *i* be the index of individuals, and *I* be the set of individuals, we have

$$f_{i}=\frac{1/c_{i}}{\sum 1/c_{i}},\;i\in I$$ (46)

where *c*_*i*_ represents the passenger cost of individual *i*, which is the value of the objective function, and it also shows the quality of the corresponding solution. To speed the process of getting an optimized solution and save calculation time, half of the parents which are better than others are added to the offspring to get a larger group and the group is sorted together for each generation, while in the standard GA, the entire population would be replaced at each iteration. After sorting, the better individuals of which the amount equals the size of the population will be chosen to be the new generation.

2. Genetic operation

Considering the standard GA, the crossover and mutation operations both focus on one random gene without any limitation, which refers to the arrival or departure time of one random service at one random station in this problem, but it is easy to get infeasible solutions for this rescheduling problem. In some previous studies, i.e., Wu et al. [32], the operator was implemented again to get one feasible solution, but it may cause low efficiency due to the complicated rules for each individual. To speed up the generation process and increase efficiency, this algorithm has introduced the feasible region for each gene, which could be calculated by the formulations and the remaining genes of this individual.

For the mutation process, the new gene that acts as the mutation result is selected randomly from the feasible region, and because there used to be a feasible solution before the mutation, the feasible region could not be empty. In this way, the children generated by mutation would always be feasible.

For the crossover strategy, genes which represent all the arrival and departure times of one random train service, for instance, $a_{f_{k-1},1},d_{f_{k-1},1},\ldots ,a_{f_{k-1},N},d_{f_{k-1},N}$, are chose and exchanged all at once. A **check process** is applied to ensure the feasibility of the children generated from crossover by checking whether all the exchanged genes are in their new feasible regions. The check process would follow the order for the chosen train service from its beginning station to the end station. If there is any invalid gene, the revision method is to find a new gene randomly in the feasible region to replace it, and the process ends when the children are feasible.

There are two conditions for the termination of the genetic algorithm. One is that the difference between the best fitness of adjacent ten iterations is less than a given value, which is represented by *ε*, for ten times consecutively, and the other is a maximum number of iterations. If the first condition is met, the algorithm terminates and the best individual of the last generation would be output as the optimized solution. Otherwise, the algorithm would continue until reaching the maximum number of iterations.

**Fig 5:**
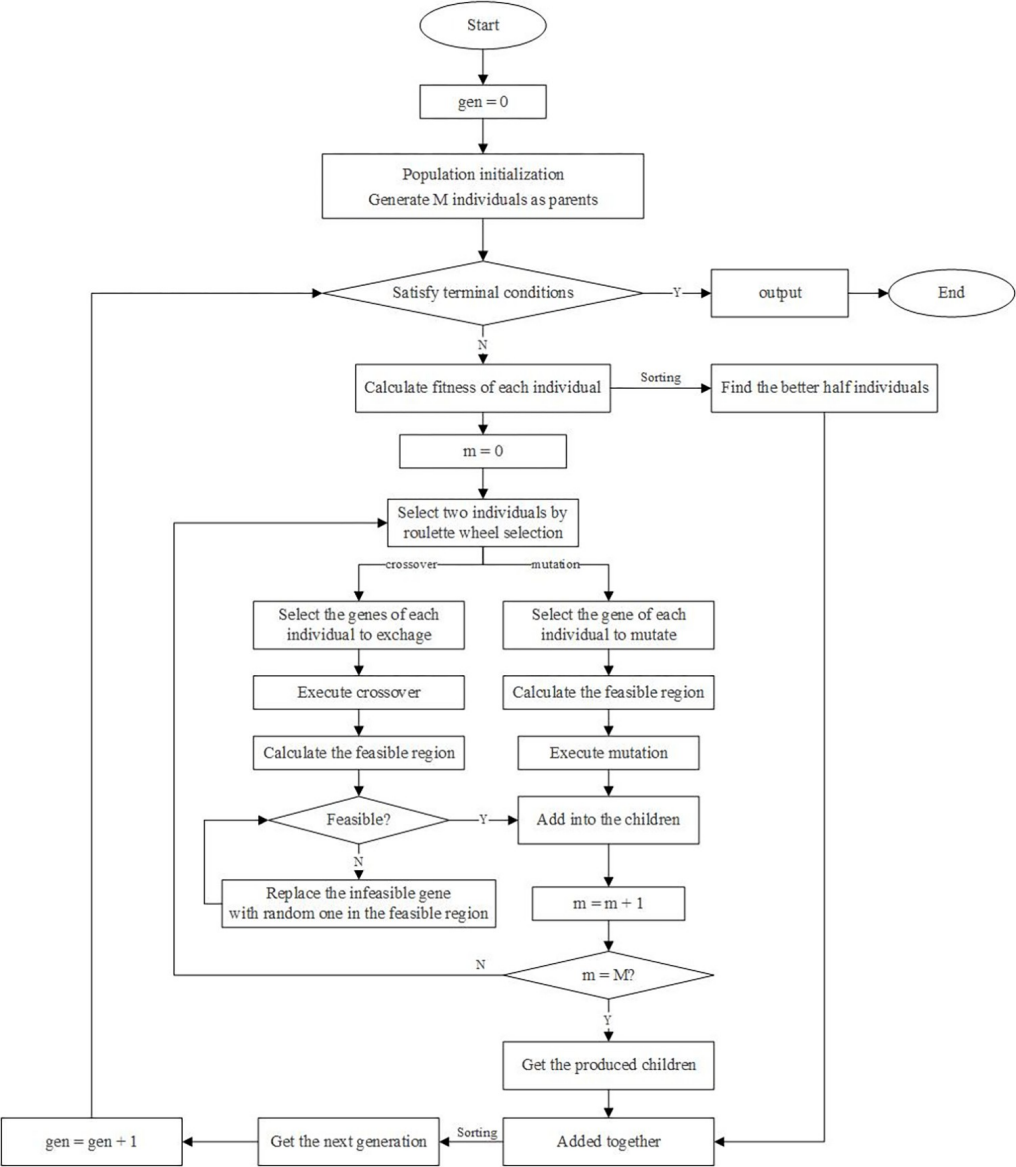
The procedure of the customized genetic algorithm.

The procedure of the customized genetic algorithm is shown in Fig 5.

### Case study

#### Basic descriptions

**Fig 6:**
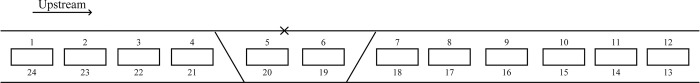
The coded chromosome of each individual.

An urban railway line of a city in China that covers 12 stations with a total length of 14.23 km is taken as the case in this work, which is shown in Fig 6. The disruption is assumed to occur at Station 5, causing the blocked section from station 5 to station 6, and two crossovers used in ADM are shown in this figure.

**Fig 7:**
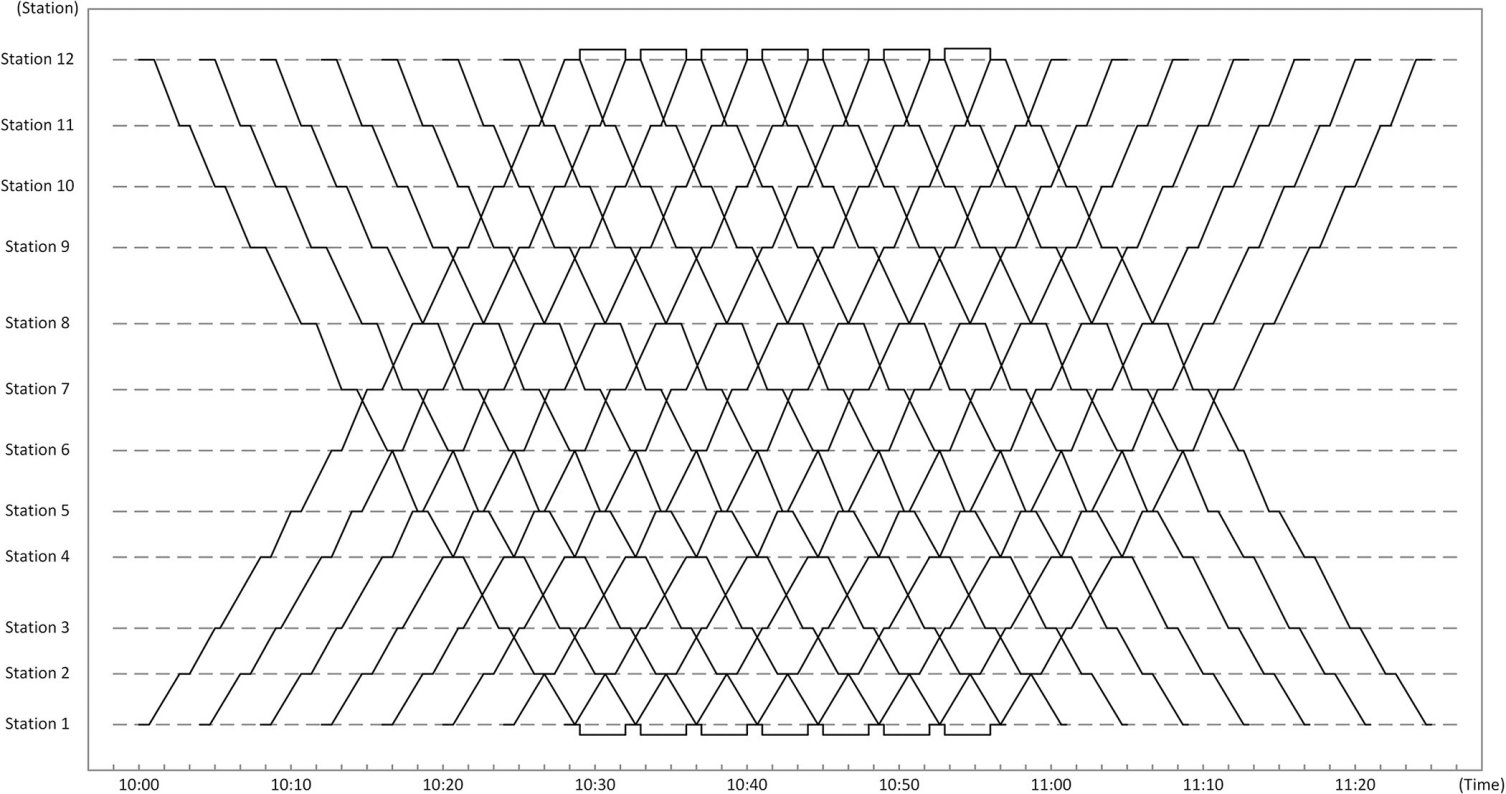
The original timetable without the disruption.

The original timetable gotten from the real operation situation is input as one part of the studied scenario and is shown in Fig 7. The studied rescheduling period is from 10:00 am to 11:25 am, which is during off-peak hours, as mentioned in *Assumption 4*, and the disruption starts at 10:15 am and ends at 10:35, with the duration 20 min for the base scenario. The planned headway between train services of the original timetable is 240s, and the number of involved train services in each direction is 15. The number of passengers comes from the practical AFC data during the studied period. Other parameters of this base scenario are provided in Table 3, and the maximum and minimum running times of each section are given in Table 4. For the maximum running times, they are calculated by adding 10s to the planned running times of the original timetable, which is according to Wang et al. [33]. While for the minimum running times, they are calculated by the section lengths and the maximum velocity of trains. Moreover, the minimum dwell time at each station is set as 20s.

**Table 3:** Parameters of the basicscenario.

Notations	Description	Value
Parameters for timetable		
*t*_*c*_	Additional time for train services to pass the crossover	100(s)
$t_{min}^{b}$	Minimum time for one rolling stock to finish the turnaround task	120(s)
*t* ^*O*^	Tolerance time of passengers	300(s)
*h*_1_	Minimum headway for two consecutive train services to arrive at or depart from one station	120(s)
*h*_2_	Minimum headway for the following service to arrive at a station that the leading one departed from	60(s)
*t*_*s*_	Security time for train services in different directions to enter the single-track section	60(s)
*t*_*p*_	Penalty time for leaving passengers	30(min)
*μ*	Coefficient of passenger feeling sensitivity	0.05
Parameters for GA		
*R*	The population size	20
*g*_*m*_	The maximum generation	2000
*p*_*c*_	the crossover probability	0.2
*p*_*m*_	the mutation probability	0.8
*ε*	the given value for termination	0.01%

**Table 4:** Bounds of running time of sections.

Section	Range of running time (s)	`Section	Range of running time (s)
1–2	[60,130]	13–14	[60,110]
2–3	[60,110]	14–15	[60,110]
3–4	[80,170]	15–16	[60,110]
4–5	[40,90]	16–17	[80,150]
5–6	[60,130]	17–18	[60,130]
6–7	[60,110]	18–19	[60,110]
7–8	[60,130]	19–20	[60,130]
8–9	[80,150]	20–21	[40,90]
9–10	[60,110]	21–22	[80,170]
10–11	[60,110]	22–23	[60,110]
11–12	[60,110]	23–24	[60,130]

## Results

**Fig 8:**
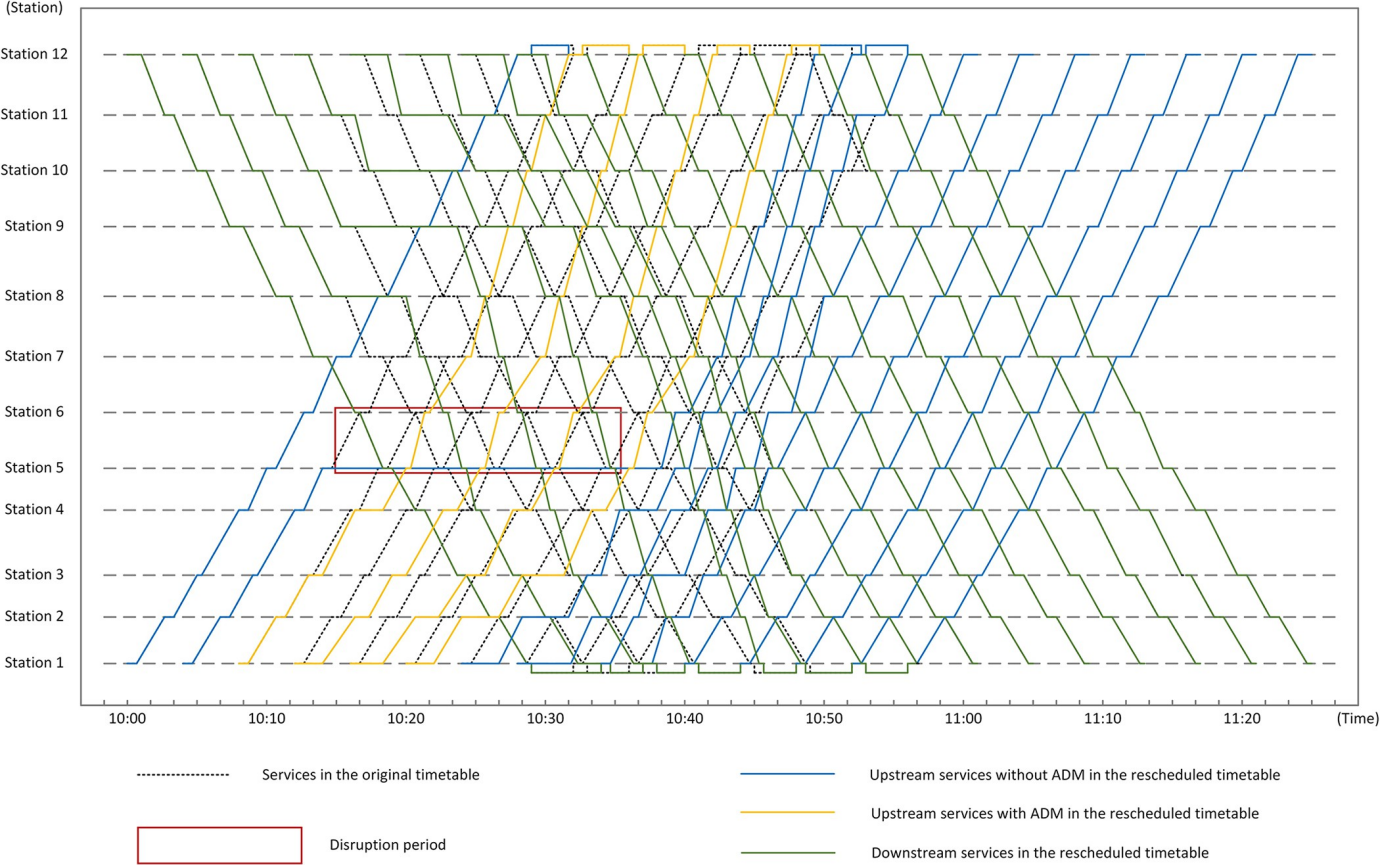
Rescheduled timetable with the rescheduling measure ADM.

**Fig 9:**
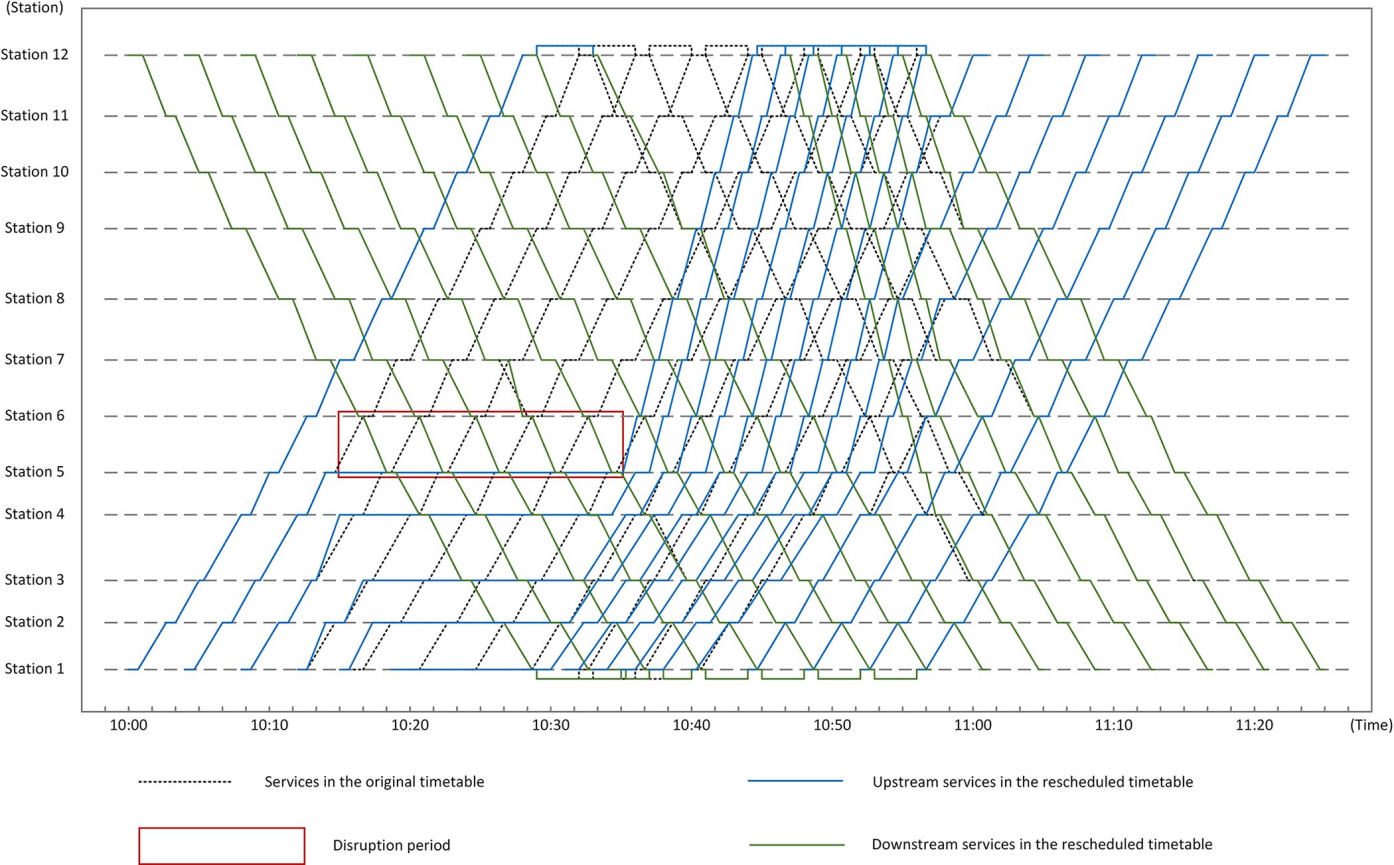
Rescheduled timetable with the practical operation measure.

The proposed model was verified by Python 3.6.5 on a PC (2.4-GHz processor speed and 8-GB memory size) with the platform Windows 10, and a rescheduled timetable was gotten as the optimized solution for the model. Figs 8 and 9 show two rescheduled timetables gotten from one experiment as an instance, which are generated by ADM and the practical operation measure separately. In Fig 8, the lines in yellow represent train services in the upstream direction which need to pass the single-line section during the disruption. In both Figs 8 and 9, the blue lines indicate upstream train services which don’t need to pass the single-line section, and the green ones show train services in the downstream direction. From Fig 8, train services in opposite directions enter the single-line section alternately during the disruption, which offers a balanced service for passengers in both directions. Moreover, the dotted lines in the two figures represent planned services in the two directions.

Specific data listed in Table 5 are the average value of ten experiments, which show the superiority of ADM in terms of a reduction of the passenger cost, namely, the improvement of passenger service quality, outperforming the practical operation measure by more than 13.5%. With applying ADM, there is a significant reduction in the service deviation, and also the ratio for passengers leaving URT declines. Each passenger would wait for nearly 10 seconds more than applying the practical operation measure, while his/her travel time is nearly 40 seconds lower.

**Table 5:** Function values by the two rescheduling measures.

Measure	*c*_*d*_ (s)	*c*_*tw*_ (s)	*c*_*ti*_ (s)	*ω* (%)	*c* (s)
ADM	2456.6	128.2	591.2	13.90	975.33
Practical Operation	4146.6	118	632	20.27	1128.09

**Fig 10:**
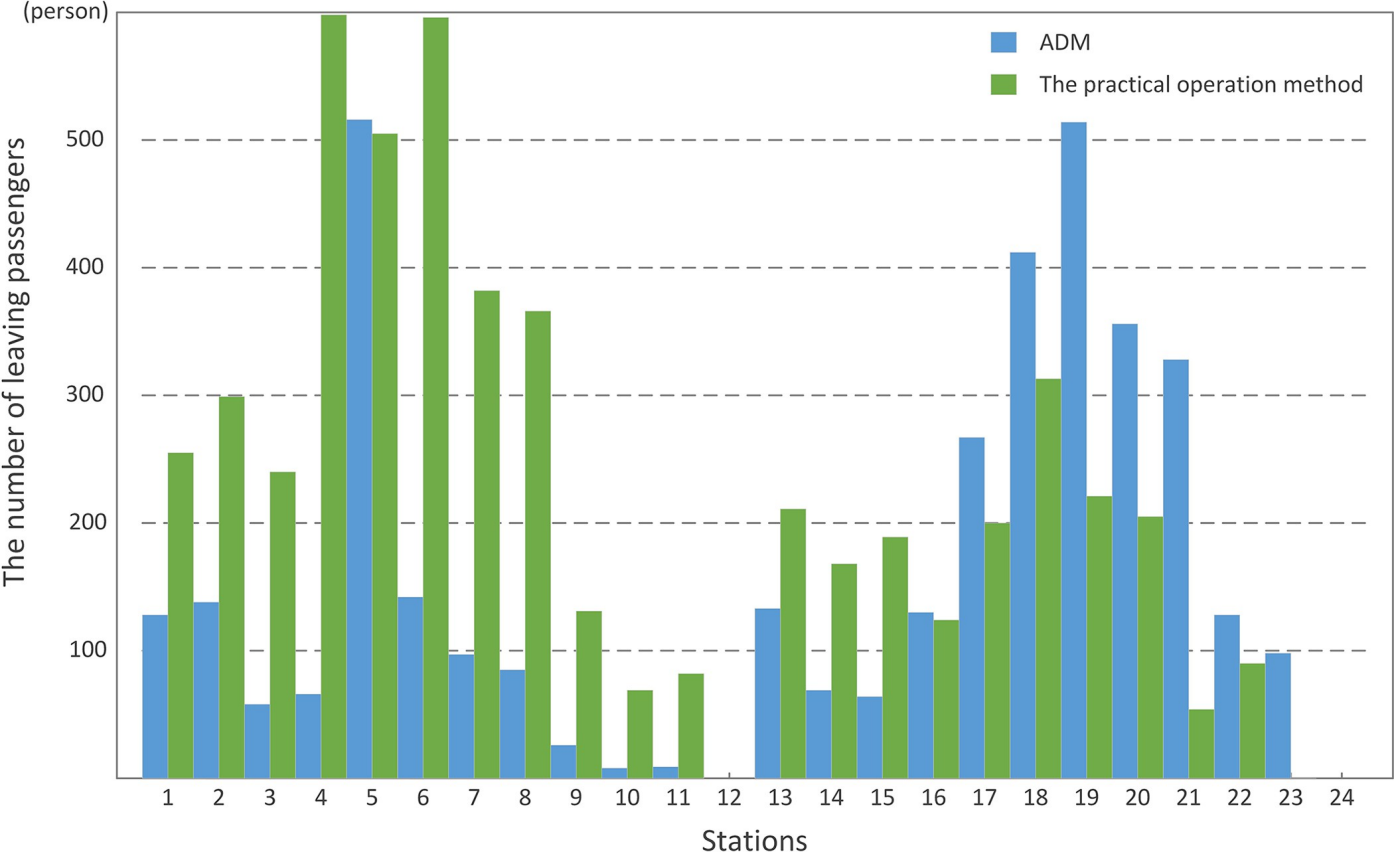
The number of leaving passengers at each station.

Besides the function values, the number of leaving passengers at each station of the line is shown in Fig 10. It could be found that applying ADM could significantly reduce the number of leaving passengers in the upstream direction, with the increased number of leaving passengers in stations 16–23. But from the whole line viewpoint, the total number of leaving passengers with ADM is 3772, which is 28.80% lower than the number 5298 with the practical operation measure.

**Fig 11:**
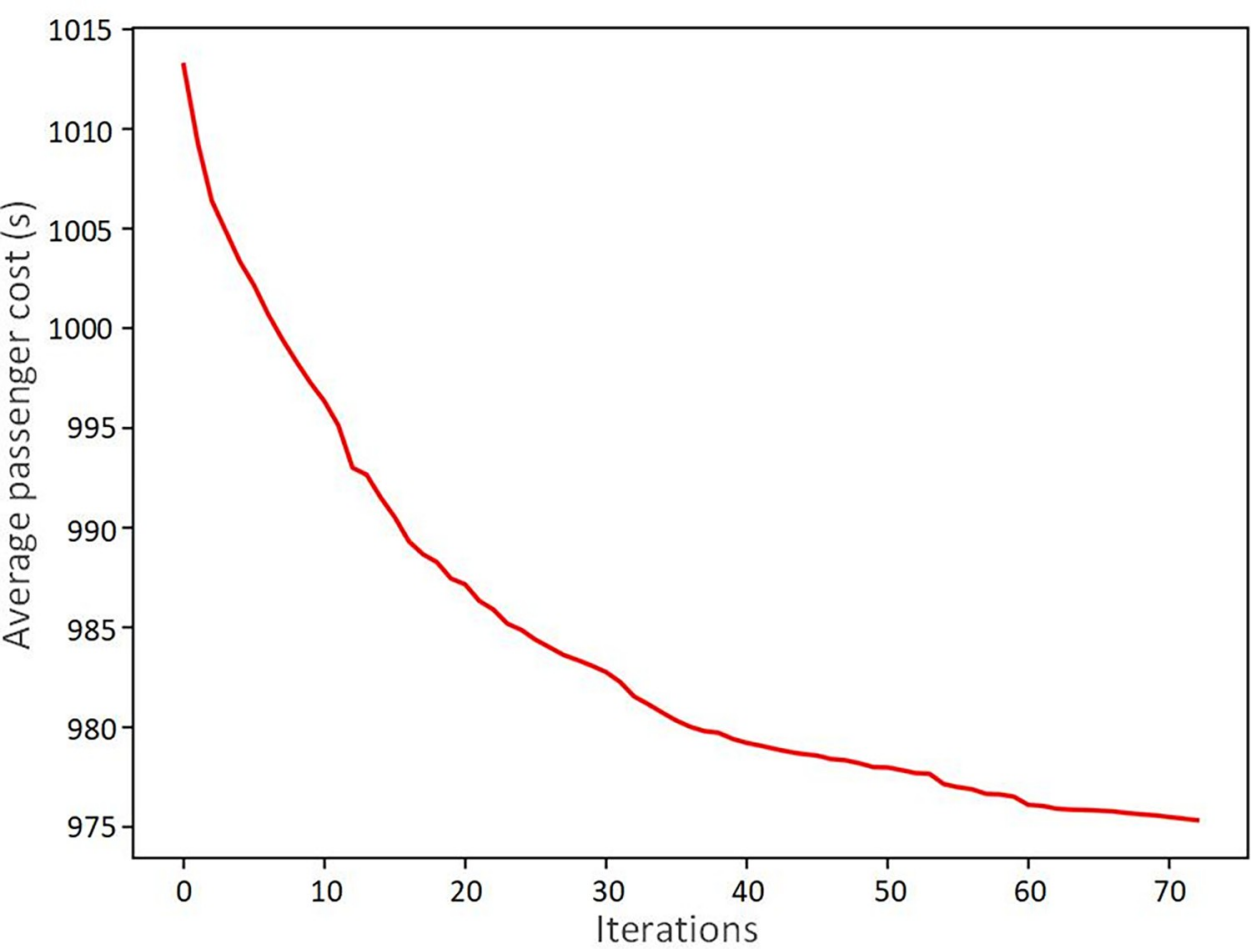
The convergence of the customized genetic algorithm.

While for the customized GA, a comparison is done in Table 6 to verify its efficiency in this rescheduling problem. Besides the standard GA, two classic metaheuristics are used to solve the proposed model, and they are the simulated annealing (SA) algorithm and the tabu search (TS) algorithm. Parameters of these algorithms are gotten from several experiments. The passenger cost, which is the value of the objective function, could indicate the solution quality of the algorithm with different improvements, and the computing time shows the efficiency of the algorithm to solve the proposed model. It could be found from Table 6 that SA shows the best computing time among the four algorithms, but it ends at the local optimal solution more easily. TS shows better performance in the solution quality compared with the standard GA as well as SA. However, the customized GA shows significant advantages in computational efficiency and solution quality compared with other three metaheuristic algorithms used for this proposed problem. Moreover, Fig 11 shows the convergence of the customized GA.

**Table 6:** Comparison of the different algorithms.

Algorithm	Passenger cost (s)	Improvement rate (%)	Computing time (s)	Improvement rate (%)
the SA	1755.23	44.43	112.45	-
the TS	1353.31	27.93	430.19	59.12
the standard GA	1582.29	38.36	386.13	54.46
the customized GA	975.33	-	175.85	-

### Sensitivity analysis

#### The coefficient of passenger feeling sensitivity

Considering the coefficient of passenger feeling sensitivity μ in the formulation (40), an analysis has been done to show how it affects the ADM rescheduling process with other parameters and conditions to be the same, and the result is shown in Table 7. The larger *μ* means that passengers are more sensitive to the deviation of timetable, namely, they could feel more unpleasant with the difference between the rescheduled timetable and the original one.

**Table 7:** Function values with different coefficients of passenger feeling sensitivity *μ*.

The value of *μ*	*c*_*d*_ (s)	*c*_*tw*_ (s)	*c*_*ti*_ (s)	*ω* (%)	*c* (s)
0.01	2898	128	572.4	14.56	885.54
0.02	2896	128.2	578.8	14.13	911.21
0.05	2456.6	128.2	591.2	13.90	975.33
0.1	2406	127.2	596	14.83	1087.91

From Table 7, with the increased value of *μ*, the travel time and the passenger cost increase, and there is a decline in the deviation of the rescheduling service pattern from the original one. It could be found that after *μ* = 0.05, the decline speed of the deviation would slow down with the value of *μ* increasing. This may be because though passengers would feel more unpleasant, fewer deviations could be eliminated except those that couldn’t be reduced due to the rescheduling constraints.

### Headway of two consecutive trains

**Fig 12:**
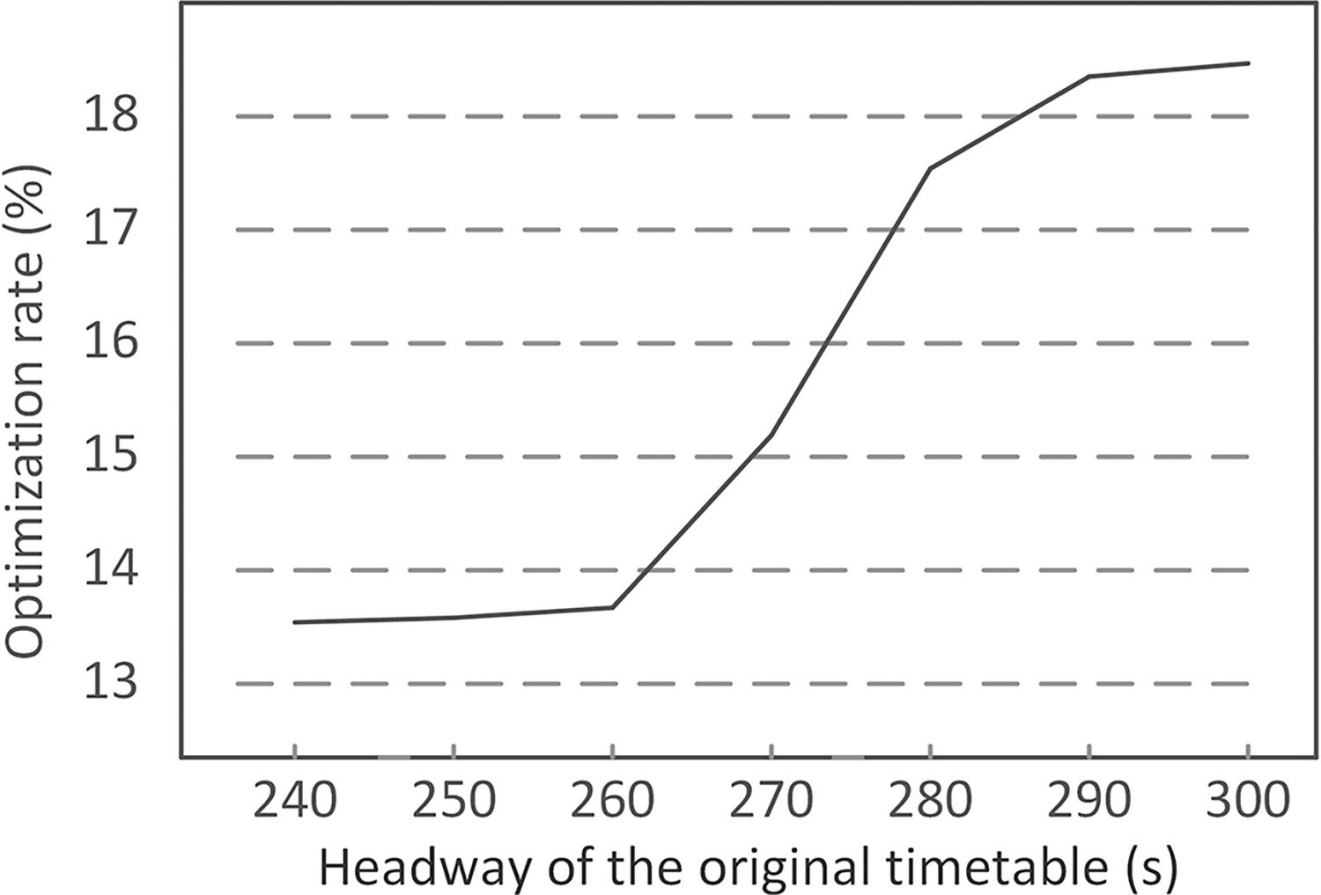
Variation of optimization rate with different headways in the scheduled pattern.

In this study, the headway of the original timetable is 240s, which is during off-peak hours and set as a parameter. Fig 12 shows the variation of optimization rate with different headways of the original timetable, comparing ADM with the practical operation measure. The optimization rate means the relative difference between the passenger cost of ADM and that of the practical operation measure, i.e., it is calculated by “(the passenger cost of the practical operation measure—the passenger cost of ADM) / the passenger cost of the practical operation measure”.

It could be found from Fig 12 that for each analyzed headway, ADM shows more than 13% advantage in reducing the passenger cost compared with the practical operation measure, meaning that ADM is better than the practical operation method with these original headways. For the basic scenario in which the disruption duration is 20 min, the optimization rate of ADM grows with the increase of planned headway, showing that the proposed method may have more advantage on the original timetable with more buffer time.

### Disruption duration

**Fig 13:**
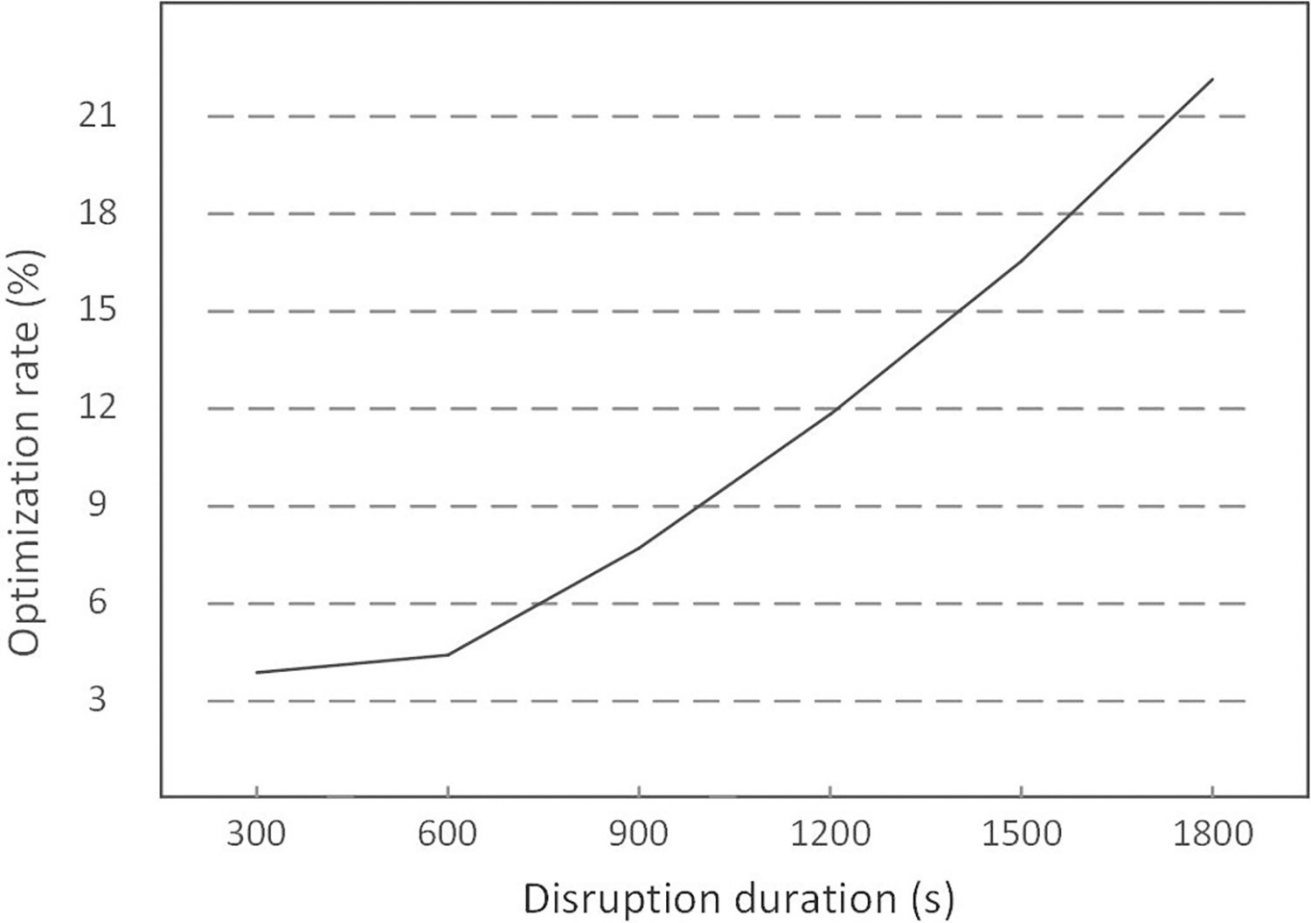
Variation of the optimization rate with different disruption durations.

In this paper, disruption duration is taken as one given parameter. Then a comparison is made between the passenger cost of ADM and that of the practical operation measure with different lengths of the disruption period, and the results are shown in Table 8. Fig 13 shows the variation of the optimization rate with different disruption durations.

**Table 8:** Sensitivity analysis of disruption duration.

Disruption duration (s)	Passenger cost (s)		Optimization rate
	ADM	Practical operation	
300	872.30	907.51	3.88%
600	892.44	933.66	4.42%
900	917.24	1012.82	9.43%
1200	975.33	1128.09	13.54%
1500	1065.34	1271.93	16.24%
1800	1124.57	1444.29	22.14%

From Table 8 and Fig 13, with the increased disruption duration, the passenger cost of both ADM and the practical operation measure would increase, as well as the increasing optimization rate, meaning that ADM shows better efficiency with a longer disruption duration. When the duration is less than 600s, namely, for disruptions under 10 minutes, there is little difference between ADM and the practical operation measure, as the optimization is less than 5%.

### Tolerable waiting time

**Fig 14:**
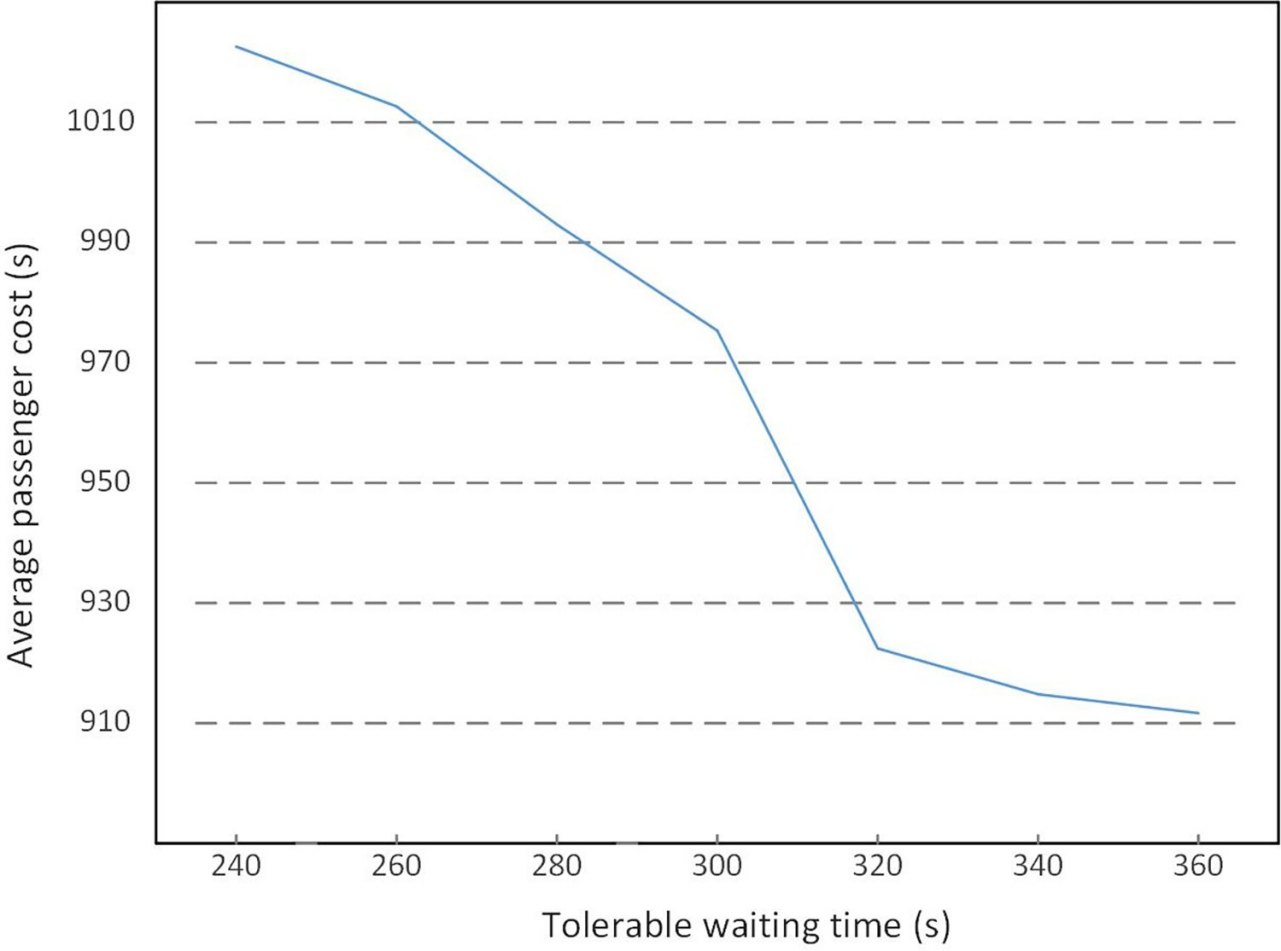
Variation of function values by ADM with different tolerable waiting times.

Tolerable waiting time, which should be a variable resulting from personal character and the travel purpose in the practical research, is simplified in this study as a constant. And with the scheduled headway of 240s, the tolerable waiting time is analyzed from 240s to 360s in Table 9, and the variation of passenger cost is shown in Fig 14.

**Table 9:** Sensitivity analysis of tolerable waiting time.

Tolerable waiting time(s)	*c*_*d*_ (s)	*c*_*tw*_ (s)	*c*_*ti*_ (s)	*ω* (%)	*c* (s)
240	2361.4	117.6	602.2	19.20	1022.61
260	2697	120	589.8	17.58	1012.65
280	2522	124.2	592.8	15.66	992.99
300	2456.6	128.2	591.2	13.90	975.33
320	2720.6	131.4	581.4	7.77	922.45
340	2608	132.2	584.2	7.13	914.82
360	2628	133.2	588.6	6.17	911.68

From Table 9 and Fig 14, the increase in the tolerable waiting time leads to a significant decrease in the leaving ratio, and the passenger cost also declines with the increased tolerable waiting time, which means improvements in passenger service quality. Tolerable waiting time could be further studied by taking the passenger difference into account, and the analysis of the tolerable waiting time could contribute to the work of leading passengers under disruptions.

### Parameters for the algorithm

As mentioned in Table 3, there are several parameters set for the proposed algorithm. Experiments are taken to analyze different combinations of parameters for GA, and the chosen parameters are the population size *R*, the mutation probability *p*_*m*_ as well as the given value for termination *ε*. Results of different combinations are shown in Table 10 as follows.

**Table 10:** Results of different combinations of the parameters for the algorithm.

Experiments	*R*	*p*_*m*_	*ε*	Passenger cost (s)	Computing time (s)
Basic scenario	20	0.8	0.01%	975.33	175.85
1	20	0.8	0.005%	968.97	339.87
2	20	0.8	0.05%	997.73	168.26
3	20	0.8	0.1%	1006.02	159.02
4	20	0.5	0.005%	986.58	341.24
5	20	0.5	0.01%	994.56	176.26
6	20	0.5	0.05%	1004.53	171.47
7	20	0.5	0.1%	1007.48	165.51
8	20	0.2	0.005%	999.47	358.62
9	20	0.2	0.01%	1003.68	176.07
10	20	0.2	0.05%	1009.90	173.18
11	20	0.2	0.1%	1012.62	167.58
12	40	0.8	0.01%	969.02	425.13
13	40	0.5	0.01%	992.25	307.70
14	40	0.2	0.01%	1000.33	317.70
15	10	0.8	0.01%	993.43	111.06
16	10	0.5	0.01%	1016.51	107.21
17	10	0.2	0.01%	1020.69	109.73

From Experiments 1–3, 4–7 and 8–11, it could be found that with other parameters to be the same, when the given value for termination *ε* is larger, the passenger cost would be larger and the computing time would be less. For the experiments of which *ε* = 0.01%, the computing time is acceptable with better solutions, while Experiments 1, 4, 8 which have *ε* = 0.005% could get relatively better solutions than other *ε* with quite longer computing times. Therefore, we prefer to using *ε* = 0.01% in the case study as well as other experiments. Also, results of these experiments show that the lower mutation probability would lead to local optimal solution more easily for the proposed problem. This may be because the applied crossover strategy would choose one train service to be exchanged, while for younger children it would be more possible for them to have same times of some train service, namely, crossover may make no changes at all. That may be why larger mutation probability could help to avoid getting local optimal solution early. For Experiments 12–17 with the same *ε* = 0.01%, different population sizes are analyzed with different mutation probability. The results show that larger population size would cause more computing time, while smaller population size would get local optimal solutions within significantly short time. Because larger population size means that each generation more feasible solutions need to be generated, which requires much more computing time, while more children could have more possibilities and help to avoid local optimal solutions.

## Conclusions

In this paper, a mixed-integer nonlinear model is developed with the rescheduling measure ADM to solve the problem with a partial blockage caused by the disruption. The passenger cost is used as the objective function, which quantifies the passenger service quality of URT. The model is solved by a customized GA to get an optimized solution in a short time, and real-world data of one subway line in China is studied in the case study to verify and analyze the advantage of ADM in promoting passenger service quality compared with the practical operation method. The results validate the proposed model and algorithm, and it is confirmed by a sensitivity analysis that with different original headways during off-peak hours, ADM is always feasible for rescheduling under partial blockage and better than the practical operation method. For the determined disruption duration, ADM has the most effective performance at one specific original headway, and for headways longer than it, the optimization rate would decline for the better performance of the practical operation method. Other sensitivity analyses have been studied, and the result of analyzing the disruption duration shows that ADM may be more adaptive for the scenario with long disruption durations. The effect on improving the passenger service quality of ADM is positively related to the tolerable waiting time of passengers, but negatively related to the sensitivity of passenger feeling about the deviation.

The presented work contributes to extending the research using ADM to deal with partial blockage in URT, and compared with related studies, time-varying passenger flow and the turnaround process are firstly taken into consideration in this paper. Nevertheless, it could be studied to integrate short-turning and ADM as intelligent rescheduling measures for disruptions, and the rescheduling pattern for each train could be more flexible in the real-time rescheduling process. Furthermore, the tolerable waiting time could be set as a variable for different groups of passengers and combined with passenger behavior analysis. Moreover, this work only considers the application of ADM to normal urban railway lines. However, it could be a new challenge to adopt such kind of flexible routing to the train rescheduling problem in URT network, since the location of disruption and the impact of transfer passenger flows need to be incorporated into the current model. Also train services in different lines may share their rolling stocks in a network problem, which is another interesting research direction in the future.

## Supporting information

S1 Table Original timetable. (XLSX)

S2 Table Passenger OD demand. (XLSX)
